# Morphological variability is greater at developing than mature mouse neuromuscular junctions

**DOI:** 10.1111/joa.13228

**Published:** 2020-06-13

**Authors:** Aleksandra M. Mech, Anna‐Leigh Brown, Giampietro Schiavo, James N. Sleigh

**Affiliations:** ^1^ Department of Neuromuscular Diseases, UCL Queen Square Institute of Neurology University College London London UK; ^2^ UK Dementia Research Institute University College London London UK; ^3^ Discoveries Centre for Regenerative and Precision Medicine University College London Campus London UK

**Keywords:** epitrochleoanconeus, fast twitch, flexor digitorum brevis, lumbricals, morphology, motor neuron, muscle fibre type, neuromuscular junction, NMJ‐morph, slow twitch, synapse, transversus abdominis

## Abstract

The neuromuscular junction (NMJ) is the highly specialised peripheral synapse formed between lower motor neuron terminals and muscle fibres. Post‐synaptic acetylcholine receptors (AChRs), which are found in high density in the muscle membrane, bind to acetylcholine released into the synaptic cleft of the NMJ, thereby enabling the conversion of motor action potentials to muscle contractions. NMJs have been studied for many years as a general model for synapse formation, development and function, and are known to be early sites of pathological changes in many neuromuscular diseases. However, information is limited on the diversity of NMJs in different muscles, how synaptic morphology changes during development, and the relevance of these parameters to neuropathology. Here, this crucial gap was addressed using a robust and standardised semi‐automated workflow called NMJ‐morph to quantify features of pre‐ and post‐synaptic NMJ architecture in an unbiased manner. Five wholemount muscles from wild‐type mice were dissected and compared at immature (post‐natal day, P7) and early adult (P31−32) timepoints. The inter‐muscular variability was greater in mature post‐synaptic AChR morphology than that of the pre‐synaptic motor neuron terminal. Moreover, the developing NMJ showed greater differences across muscles than the mature synapse, perhaps due to the observed distinctions in synaptic growth between muscles. Nevertheless, the amount of nerve to muscle contact was consistent, suggesting that pathological denervation can be reliably compared across different muscles in mouse models of neurodegeneration. Additionally, mature post‐synaptic endplate diameters correlated with fibre type, independently of muscle fibre diameter. Altogether, this work provides detailed information on healthy pre‐ and post‐synaptic NMJ morphology from five anatomically and functionally distinct mouse muscles, delivering useful reference data for future comparison with neuromuscular disease models.

## Graphical abstract

Using a semi‐automated workflow called NMJ‐morph to quantify features of neuromuscular junction (NMJ) architecture across several different muscles and timepoints, Mech *et al*. reveal that post‐synaptic morphology is more variable between muscles than the pre‐synaptic structure of innervating motor neuron terminals. Moreover, the developing NMJ shows greater diversity than the mature synapse, perhaps caused by the observed distinctions in synaptic growth.

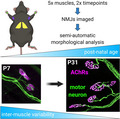

## 1 INTRODUCTION

The neuromuscular junction (NMJ) is a peripheral synapse formed between lower motor neurons and skeletal muscle fibres that has been studied in many different species, including humans (Nyström, 1968; Kuno *et al*., 1971; Cramer and Van Essen, 1995; Prakash *et al*., 1996; Campbell and Ganetzky, 2012; Sakowski *et al*., 2012; Hammond *et al*., 2016; Jones *et al*., 2017; Grice *et al*., 2018). Upon action potential‐mediated depolarisation of the pre‐synaptic neuronal membrane, the neurotransmitter acetylcholine (ACh) is released into the NMJ synaptic cleft, where it binds to ACh receptors (AChRs) found in high density on post‐synaptic muscle membranes in direct apposition to motor nerve terminals. This results in sodium ion influx into the muscle, post‐synaptic membrane depolarisation and, if a threshold is reached, excitation−contraction coupling and muscle contraction. Due to its experimental accessibility and large size, the NMJ has been used for many years as a model for pre‐ and early post‐natal synapse development (Sanes and Lichtman, 1999). During this time, the mammalian NMJ undergoes a series of growth and maturation processes that refine the synaptic architecture and enhance neurotransmission efficiency to facilitate motor performance (Shi *et al*., 2012).

At birth, mammalian NMJs are poly‐innervated, meaning that the endplate is contacted by more than one motor nerve ending, and in some instances more than 10 different axons (Tapia *et al*., 2012). During early post‐natal development, equating to less than 2 weeks in mice, asynchronous axon withdrawal occurs and NMJs become mono‐innervated. Known as synapse elimination, this process is thought to refine peripheral nerve architecture, and is driven by activity, inter‐nerve competition and reciprocal nerve−muscle−glia signalling (Favero *et al*., 2012; Turney and Lichtman, 2012; Smith *et al*., 2013b). This is accompanied by significant changes in the post‐synapse to maintain the close proximity of motor terminals to AChRs. For example, the density and number of AChRs increase, and the morphology of AChR clusters transforms from a simple, plaque‐like shape to a complex, multi‐perforated, pretzel‐like structure (Marques *et al*., 2000). This is, at least in part, due to remodelling of the post‐synaptic membrane to create junctional folds, enlarging the contact area within the synaptic cleft. Moreover, the molecular composition of the membrane also changes to assure correct functioning of the mature synapse; for instance, the foetal γ AChR subunit is exchanged for the adult ε subunit (Missias *et al*., 1996).

Mammalian muscles are composed of two main classes of force‐generating (extrafusal) muscle fibre, ‘slow twitch’ (type I) and ‘fast twitch’ (type II), which have differing anatomical, physical and metabolic properties (Schiaffino and Reggiani, 2011). Slow twitch fibres generate energy through oxidative metabolism and can be continuously used for long periods of time (i.e. hours), therefore they function primarily to maintain body posture. Based on differential expression of myosin heavy chain (MHC) genes, fast twitch fibres can be further subdivided into three principal categories: type IIa (expressing *MYH2*); IIx (expressing *MYH1*); and IIb (expressing *MYH4*; Talbot and Maves, 2016). The time to contraction and time to fatigue of fast twitch fibres is shorter than slow twitch, and they lie on a spectrum of decreasing times from IIa to IIb. Type IIa fibres are oxidative and most similar to slow twitch fibres, while type IIx and IIb are both glycolytic, with IIb fibres being required for the shortest and most intense movements. There are conflicting reports as to size categories of each fibre subtype, which is probably due to their diameters fluctuating between types of muscle; for example, slow twitch fibres are larger than fast twitch in the rat soleus muscle, which is composed of 86%–93% slow twitch fibres, but smaller in the rat extensor digitorum longus (EDL) muscle that is made up of only 2%–3% slow twitch fibres (Alnaqeeb and Goldspink, 1987). Nevertheless, slow twitch fibres are more often reported as being smaller than fast twitch, with type IIb being the largest (Stifani, 2014). In addition to intrinsic disparities, muscle fibre types are innervated by distinct classes of alpha motor neuron (Stifani, 2014), the firing patterns of which appear to impact the contractile properties of fibres (Pette and Vrbová, 1985), while the fibre also appears to be able to retrogradely influence the identity of the innervating neuron (Chakkalakal *et al*., 2010). The motor neurons that innervate slow twitch fibres generally have smaller cell bodies and axons, require a lower threshold to stimulation, and have slower conduction speeds, whereas fast‐fatiguable motor neurons innervating type IIb fibres are at the other end of the spectra for these characteristics (Stifani, 2014).

The size of NMJs has been reported to correlate with fibre diameter in several different species and muscles (Harris, 1954; Nyström, 1968; Kuno *et al*., 1971; Balice‐Gordon and Lichtman, 1990; Sieck et al., 2012). Moreover, manipulations of muscle fibre size can cause equivalent changes in the NMJ (Balice‐Gordon *et al*., 1990). However, the relationship between size of NMJs and fibres may only be observable when fibre classes are individually assessed (Waerhaug and Lømo, 1994; Prakash *et al*., 1995a) or within certain types of muscle (Jones *et al*., 2016). Indeed, similar to muscle fibre diameters, inconsistencies in morphological features of NMJs associated with the distinct fibre types have been reported. For instance, using both light and electron microscopy, the average area of NMJs in predominantly slow twitch soleus muscles of the rat was shown to be larger than in the fast twitch EDL (McArdle and Sansone, 1977; Wood and Slater, 1997). Contrasting with this, NMJs innervating type I fibres in rat diaphragm muscle were shown to be less than half the size of those innervating type II fibres (Prakash *et al*., 1996). However, correcting for fibre diameter, the NMJ size does not vary quite so much across the fibre types and muscles, although type I fibre NMJs are larger than fast twitch NMJs in rat diaphragm muscle (Prakash *et al*., 1995b, 1996; Prakash and Sieck, 1998). This suggests that features other than fibre diameter contribute to NMJ size differences between muscle fibre types, for example, the innervating motor neuron and its firing properties (Waerhaug and Lømo, 1994).

The NMJ is an early and important site of pathology in many different neuromuscular conditions, including amyotrophic lateral sclerosis (Williamson *et al*., 2019), spinal muscular atrophy (Murray *et al*., 2008) and Charcot‐Marie‐Tooth disease (Spaulding *et al*., 2016). Furthermore, aging appears to result in structural alterations at the rodent NMJ (Gonzalez‐Freire *et al*., 2014), while axotomy causes NMJ dismantling prior to axonal degeneration (Miledi and Slater, 1970). Consequently, it is of considerable importance to be able to accurately assess the integrity of neuromuscular synapses in models of pathology. Pathological NMJ phenotypes (e.g. denervation counts and neurofilament accumulation) are often assessed by eye, possibly reducing objectivity and limiting inter‐study comparisons. However, this issue has been tackled by the Gillingwater laboratory, who have developed a free, ImageJ‐based methodology called NMJ‐morph for comparative analyses of neuromuscular synapses (Jones *et al*., 2016), which has been successfully used to evaluate NMJs in patients with cancer cachexia (Boehm *et al*., 2020). By using thresholded, binary images, data on 20 NMJ morphological variables can be quickly and reliably obtained, with the proviso that several are not independent (e.g. nerve terminal perimeter and area). Operator‐independent information on the pre‐synaptic nerve terminal, post‐synaptic AChRs and overlapping features can be objectively generated for accurate cross‐muscle, cross‐study and even cross‐laboratory comparisons.

Here, the same software was used to assess morphology of neuromuscular synapses at two different timepoints, representing a developing (postnatal day 7, P7) and a mature (P31−32) NMJ. Performing analyses in five different wholemount muscles with varying fibre type populations, this novel morphometric approach was used to assess the influence of post‐natal maturation, fibre type and fibre diameter on pre‐ and post‐synaptic NMJ morphology, as well as post‐natal synaptic development/growth.

## 2 MATERIALS AND METHODS

### 2.1 Animals

Wild‐type mice on a C57BL/6J background were killed at P7 and P31−32 for post‐natal developmental and early adult timepoints, respectively. Muscles were harvested from three males and three females at each age, weighing an average of 4.0 g at P7 and 17.0 g at P31−32 (Table S1). P7 animals were taken from a single litter, whereas P31−32 mice came from two different litters. All muscles were dissected from the left side of the body, except for lumbricals of the forepaw, which were taken from the right. Mouse handling and experiments were performed under license from the UK Home Office in accordance with the Animals (Scientific Procedures) Act (1986) and approved by the University College London – Queen Square Institute of Neurology Ethics Committee.

### 2.2 Tissue preparation and immunohistochemistry

Muscles were dissected (Murray *et al*., 2014; Sleigh *et al*., 2014a; Tarpey *et al*., 2018) and stained as previously described (Sleigh *et al*., 2014b, 2017). The following antibodies were used to co‐visualise axons and pre‐synaptic nerve terminals: 1/250 mouse anti‐neurofilament [2H3, Developmental Studies Hybridoma Bank (DSHB), Iowa City, IA, USA, supernatant] and 1/25 mouse pan anti‐synaptic vesicle 2 (SV2, DSHB, supernatant). 1/1,000 Alexa Fluor 555 α‐bungarotoxin (α‐BTX; Life Technologies, B35451) was used to identify post‐synaptic AChRs.

### 2.3 Neuromuscular junction imaging and analysis

Neuromuscular junctions were imaged using an LSM 510 laser‐scanning microscope (Zeiss, Oberkochen, Germany) with Argon/2 and HeNe543 lasers for neurons (labelled green; 2H3/SV2) and post‐synaptic AChRs (labelled red; α‐BTX), respectively. Z‐stacks with a 1‐µm interval were taken using a Plan‐Apochromat 63 ×/1.4 oil immersion objective. Images of P31−32 samples were taken with 0.7 × zoom, and images of P7 samples with 2 × zoom. ImageJ software (https://imagej.nih.gov/ij/) combined with the NMJ‐morph workflow (Jones *et al*., 2016) was used to describe 19 morphological variables of NMJs across all five muscles. The resolution of analysed images was 512 × 512 pixels, corresponding to 101.02 × 101.02 µm at P31−32 and 35.71 × 35.71 µm at P7. For all variables, 20 *en face* NMJs with clearly visible pre‐synaptic axons and terminals were assessed (resulting in analysis of 1200 NMJs in total). A 20th variable of poly‐innervation percentage was visually assessed at 100 NMJs per muscle (resulting in analysis of 6000 NMJs in total), as previously outlined (Sleigh *et al*., 2014b). In the case of lumbrical and flexor digitorum brevis (FDB) muscles, where more than one muscle was processed, NMJs were assessed across all muscles (as were muscle fibres, see below). All analyses were performed on maximum intensity Z‐stack projections, and no samples were excluded from analyses once imaged. Of several available thresholding methods, three provided the most accurate binary representation of NMJs. ‘*Huang*’, ‘*Yen*’ or manual method were chosen based on how well they resembled the original image. The ‘*Huang*’ method was used for 56% and 79% of NMJs from P31−32 and P7, respectively, the ‘*Yen*’ method for 42% and 1%, and the manual method for 2% and 20%.

For clarity, ‘endplates’, ‘AChRs’ and ‘AChR clusters’ are slightly different post‐synaptic structures of decreasing size. Endplates include the post‐synaptic staining, along with associated perforations and other areas devoid of α‐BTX that occur within the perimeter of staining. Hence, endplate area is a good proxy for overall NMJ size. AChRs represent the footprint of post‐synaptic staining alone (i.e. excluding α‐BTX‐negative areas). AChR clusters include only the α‐BTX staining found in discrete groupings.

### 2.4 Muscle fibre diameter analysis

After imaging P31−32 NMJs for morphological analyses, microscope slides were immersed in phosphate‐buffered saline to facilitate coverslip removal. Muscles were then teased using forceps to separate and aid identification of individual fibres, before re‐mounting in Fluoromount‐G on new microscope slides. Muscles were imaged at a resolution of 1024 × 1024 by differential interference contrast microscopy on an inverted LSM780 laser‐scanning microscope (Zeiss) with a 20 × objective. Using ImageJ, fibre diameters were determined by taking five width measurements at different points along individual fibres. Damaged fibres were avoided, as were endings that were patently larger than the rest of the fibre. Thirty fibres were measured per muscle per animal to generate mean values (resulting in analysis of 900 fibres in total). Analysed fibres were randomly sampled and very unlikely to be those in which NMJ morphology was assessed.

### 2.5 Statistical analysis

Data were assumed to be normally distributed unless evidence to the contrary could be provided by the D'Agostino and Pearson omnibus normality test. GraphPad Prism 8 (version 8.4.0) was used for all statistical analyses. Means ± *SEM* are plotted and, when possible, individual data points generated from each mouse (i.e. the mean values from 20 NMJs or 30 fibres). In all bar charts, muscles are plotted on the *x*‐axes in ascending order of the percentage of fast twitch fibres (Table 1). Moreover, the muscle colour scheme is maintained throughout all figures and supplementary figures [i.e. yellow, transversus abdominis (TVA); cyan, hindpaw lumbricals; purple, epitrochleoanconeus (ETA); red, FDB; green, forepaw lumbricals]. Individual morphological variables were statistically analysed using a repeated‐measures one‐way analysis of variance (anova), because the five dissected muscles were all taken from the same six mice per timepoint, i.e. the muscles were matched. If the anova indicated a significant difference, Bonferroni's multiple comparisons test was performed to assess significance between pairwise combinations of all five muscles. Correlation was assessed using Pearson's product moment correlation. When performing 20 associated tests (i.e. the number of tests used per timepoint), a value of *P* < 0.00256 was used to determine significance and thereby maintain an *α* of 0.05. To calculate the percentage difference in morphological variables between timepoints, the mean value at P31−32 was divided by the mean at P7 and multiplied by 100. Two‐way anovas were performed for each morphological trait (test variables being age and muscle), followed by Sidak's multiple comparisons test to assess whether there was a difference between timepoints in each muscle.

**Table 1:** Characteristics of the five muscles used in this study

Muscle	Body position	Innervating nerve	% Fast twitch	Animal	Reference
Epitrochleoanconeus (ETA)	Forelimb	Radial	90	Mouse	Bradley *et al*. (1989)
Flexor digitorum brevis (FDB)	Hindlimb	Medial plantar	95.6	Mouse	Tarpey *et al*. (2018)
Forepaw lumbricals	Forelimb	Median/ulnar	100	Mouse	Russell *et al*. (2015)
Hindpaw lumbricals	Hindlimb	Medial/lateral plantar	90	Mouse	Smith *et al*. (2013a)
Transversus abdominis (TVA)	Thorax	Lower intercostal	68	Rat	Delp and Duan (1996)

See also Figure 1.

### 2.6 Principal component analysis

Principal component analysis (PCA) was used for dimensionality reduction of the datasets of P7 and P31−32 NMJ morphological variables by collapsing the variables into new ‘principal components’ created through linear combinations of the original datasets. Data.table (https://CRAN.R‐project.org/package=data.table) and tidyverse (Wickham *et al*., 2019) packages were used to load the morphological data into R (version 3.6.2). PCA plots and loading analysis were created using the PCAtools package (https://github.com/kevinblighe/PCAtools) and ggplot2 (Wickham, 2016).

### 2.7 Clustering analysis

Morphological variables were converted to *z*‐scores, and samples clustered by Euclidean distance and hierarchal clustering. The complete linkage method was used to find breaks. Euclidean distance between two samples is the square root of the sum of squared differences between all *z*‐score‐scaled morphological variables. Complete clustering is a distance metric that defines the distance between clusters as the maximum possible distance between points in a cluster. Heat maps and clustering were created using the pheatmap package (https://CRAN.R‐project.org/package=pheatmap).

## 3 RESULTS AND DISCUSSION

### 3.1 Wholemount immunohistochemical analyses of mouse muscles and neuromuscular junctions

**Figure 1:**
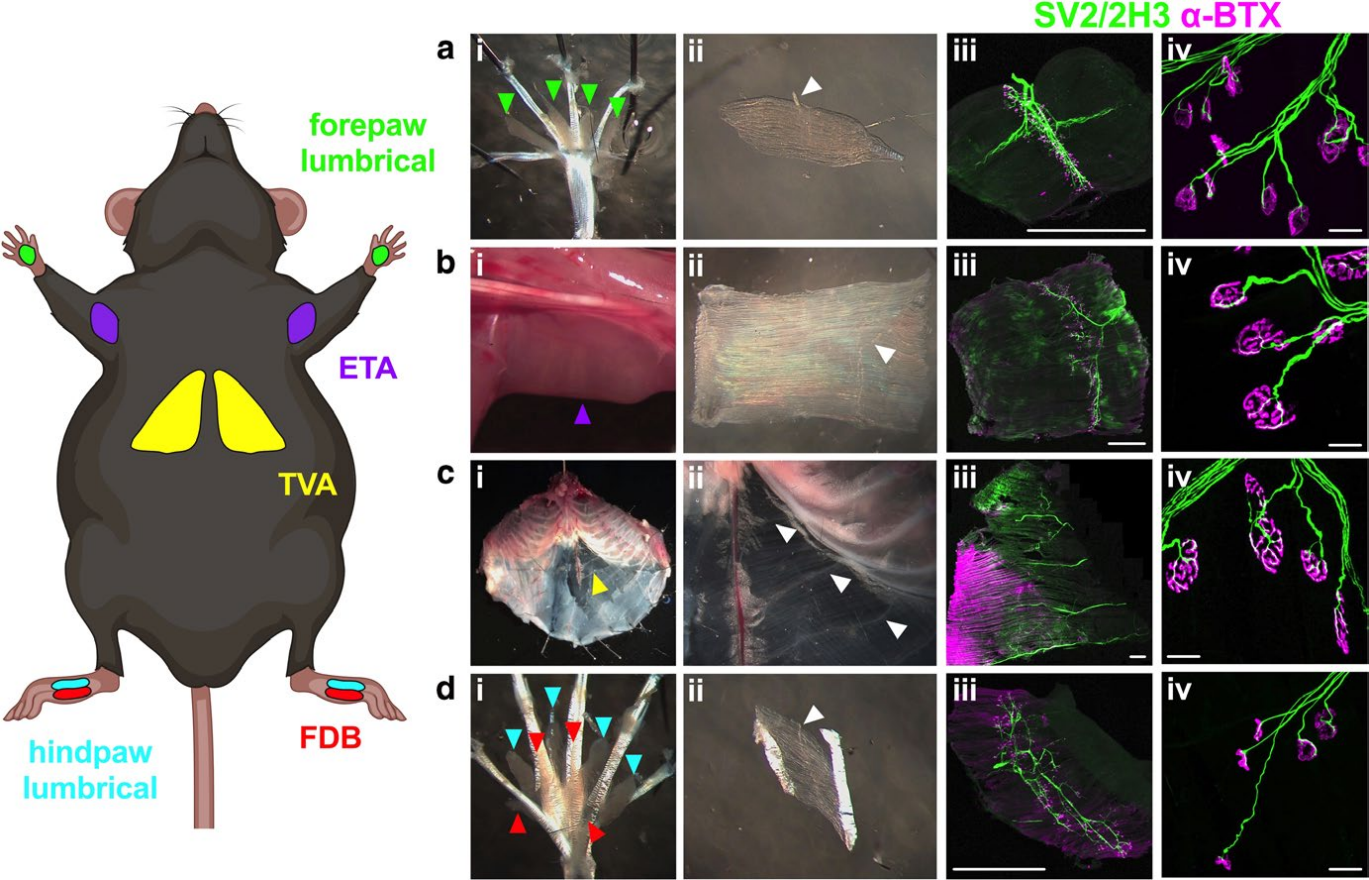
Dissection of five different muscles for wholemount assessment of neuromuscular junction (NMJ) morphology. The forepaw lumbricals (green, a), epitrochleoanconeus (ETA; purple, b), transversus abdominis (TVA; yellow, c), hindpaw lumbricals (cyan, di) and flexor digitorum brevis (FDB; red, d) are five thin and flat muscles that can be dissected from mice (i and ii) and immunohistochemically analysed as wholemount preparations (iii) to assess pre‐ (SV2/2H3, green) and post‐synaptic (alpha bungarotoxin, α‐BTX, magenta) NMJ morphology (iv). Representative staining of hindpaw lumbrical NMJs can be found in Figure 2(a). Imaged muscles were taken from a P31 mouse. Individual muscles in panel i are highlighted by arrowheads coloured the same as muscles in the mouse schematic on the left; this colour scheme is maintained in all figures and supplementary figures. White arrows in panel ii identify innervating nerves. Scale bars: 1 mm (iii); 20 µm (iv). The mouse schematic was created with BioRender (https://biorender.com). See also Table 1 [Colour figure can be viewed at wileyonlinelibrary.com]

**Figure 2:**
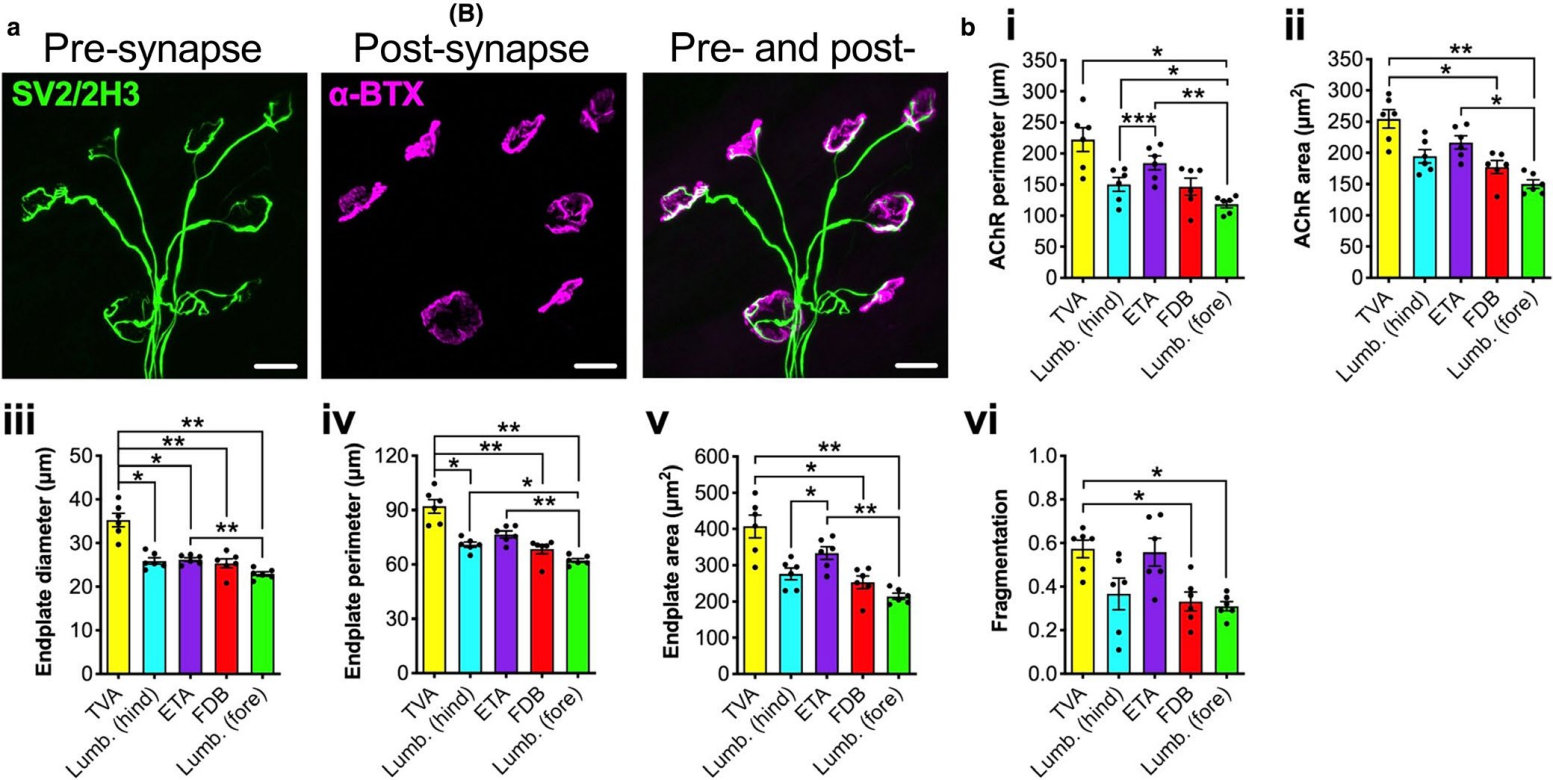
Post‐synaptic morphology of mature neuromuscular junctions (NMJs) is more variable between muscles than pre‐synaptic. (a) Five different wholemount muscles were dissected from P31−32 mice, and immunohistochemically processed to visualise and assess pre‐ (SV2/2H3, green) and post‐synaptic (α‐bungarotoxin, α‐BTX, magenta) morphology at mature NMJs. The image is of a P31 hindpaw lumbrical muscle. Scale bars: 20 µm. (b) Of 20 morphological variables analysed using repeated‐measures one‐way anovas (Table S2), only post‐synaptic features varied between muscles (only significant data are presented, i.e. Bonferroni‐corrected *P* < 0.00256). ^*^ *P* < 0.05, ^**^ *P* < 0.01, ^***^ *P* < 0.001 Bonferroni's multiple comparisons test. Means ± *SEM* are plotted (*n* = 6), as well as individual data points generated from each mouse (i.e. the mean values from 20 NMJs). *Lumb. (fore)*, lumbricals of the forepaw; *Lumb. (hind)*, lumbricals of the hindpaw. See also Tables 2 and S2 [Colour figure can be viewed at wileyonlinelibrary.com]

**Figure 3:**
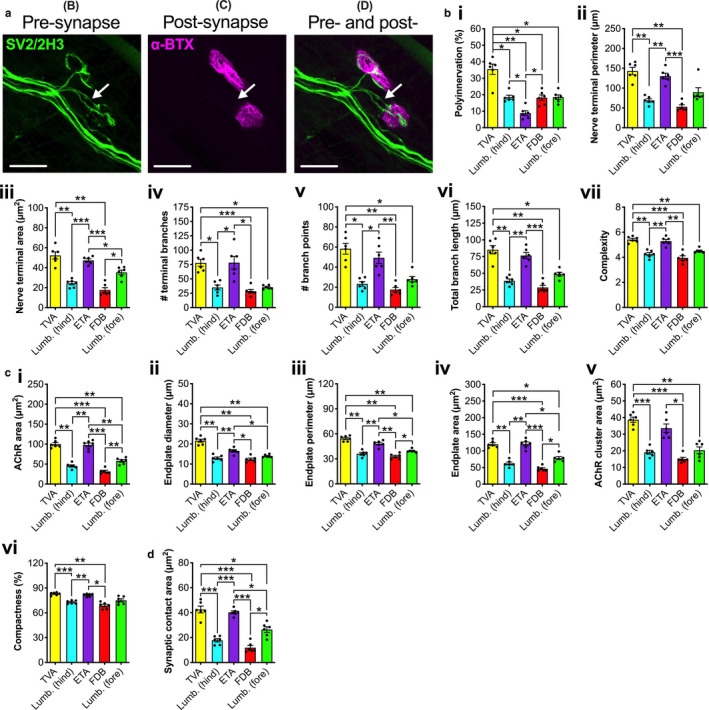
The developing neuromuscular junction (NMJ) shows greater inter‐muscular variability in morphology than mature synapses. (a) Five different wholemount muscles were dissected from P7 mice, and immunohistochemically processed to visualise and assess pre‐ (SV2/2H3, green) and post‐synaptic (α‐bungarotoxin, α‐BTX, magenta) morphology at developing NMJs. The image is of a P7 transversus abdominis (TVA) muscle, and the arrow identifies a poly‐innervated NMJ. Scale bars: 20 µm. (b–d) Of 20 morphological variables analysed using repeated‐measures one‐way anovas (Table S3), several different pre‐synaptic (b), post‐synaptic (c) and overlapping (d) morphologies varied between muscles (only significant data are presented, i.e. Bonferroni‐corrected *P* < 0.00256). ^*^ *P* < 0.05, ^**^ *P* < 0.01, ^***^ *P* < 0.001 Bonferroni's multiple comparisons test. Means ± *SEM* are plotted (*n* = 6), as well as individual data points generated from each mouse (i.e. the mean values from 20 NMJs). *Lumb. (fore)*, lumbricals of the forepaw; *Lumb. (hind)*, lumbricals of the hindpaw. See also Tables 3 and S3 [Colour figure can be viewed at wileyonlinelibrary.com]

**Figure 4:**
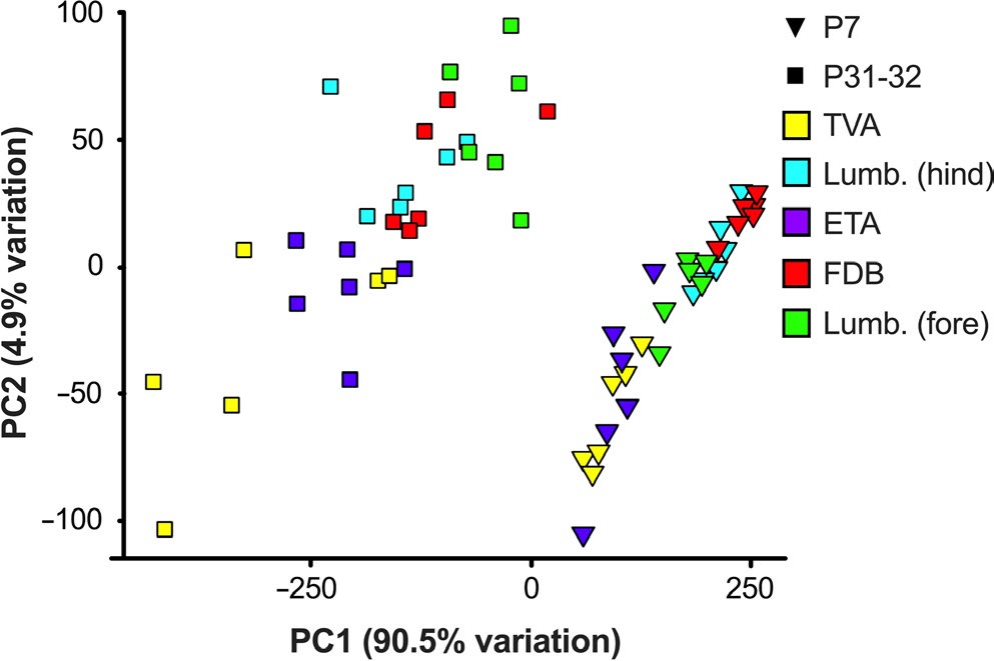
Principal components analysis (PCA) of developing and mature neuromuscular junction (NMJ) morphology. Two‐dimensional PCA representation of NMJs as defined by 20 morphological variables from each animal, colour‐coded by muscle type. Inverted triangles and squares identify P7 and P31−32 data, respectively. The first principal component (PC1, *x*‐axis) accounts for 90.5% of the variability, and PC2 (*y*‐axis) 4.9%. Each datum represents the centre point of the map positions of averages taken from 20 NMJs for each muscle from each animal. Note the ability of PC1 to distinguish between timepoints and PC2 to generally separate muscle types. *Lumb. (fore)*, lumbricals of the forepaw; *Lumb. (hind)*, lumbricals of the hindpaw. See also Figure S1 [Colour figure can be viewed at wileyonlinelibrary.com]

**Figure 5:**
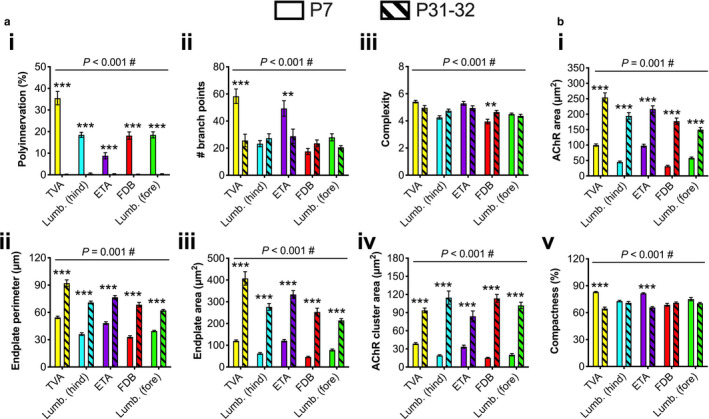
Post‐natal neuromuscular junction (NMJ) development varies between muscles. (a and b) To assess the change in morphological variables from P7 (clear bars) to P31−32 (hatched bars), values from the five muscles at both timepoints were analysed using two‐way anovas (age and muscle as the test variables; Table S4). Of the 20 variables, three pre‐synaptic (a) and five post‐synaptic (b) displayed significant interactions (#Bonferroni‐corrected *P* < 0.00256), suggesting that NMJs mature differentially between muscles. Means ± standard error of the mean are plotted (*n* = 6). ^**^ *P* < 0.01, ^***^ *P* < 0.001 Sidak's multiple comparisons test. *Lumb. (fore)*, lumbricals of the forepaw; *Lumb. (hind)*, lumbricals of the hindpaw. See also Table S4 [Colour figure can be viewed at wileyonlinelibrary.com]

Five mouse muscles from diverse body positions with varying innervating nerves and fast twitch fibre percentages (Table 1), all of which are also found in humans, were selected for morphological analysis of NMJs (Figure 1). The lumbricals of the forepaw mediate metacarpophalangeal joint flexion (i.e. paw clasping), are innervated by the median/ulnar nerve, and are composed of ~100% fast twitch muscle fibres (Figure 1a; Russell *et al*., 2015). Consisting of ~90% fast twitch fibres and innervated by the radial nerve, the ETA is located on the medial surface of the upper forelimb and contributes to forearm supination (i.e. outward rotation of the palm; Figure 1b; Bradley *et al*., 1989). Innervated by lower intercostal nerves, the TVA is a postural muscle also involved in respiration that is found in the anterior abdominal wall consisting of ~68% fast twitch fibres (Figure 1c; Delp and Duan, 1996; Murray *et al*., 2014). Situated in the hindpaw, the FDB and lumbricals aid metatarsophalangeal joint extension (i.e. paw opening) and flexion (i.e. paw clasping), respectively (Figure 1d). The FDB consists of ~96% fast twitch fibres and is innervated by the medial plantar nerve (Tarpey *et al*., 2018), whereas the hindpaw lumbricals are ~90% fast twitch and innervated by both medial and lateral plantar nerves (Smith *et al*., 2013a). All five muscles possess the major advantage that they are thin and flat, and can, therefore, be dissected and immunohistochemically processed as wholemount preparations without the need for sectioning (Figure 1, 2, 3, 4, 5, 6i1, 2, 3, 4, 5, 6ii). This permits visualisation of the entire innervation pattern of these muscles using combined neuron‐specific SV2/2H3 antibodies and AChR‐targeting α‐BTX (Figure 1, 2, 3, 4, 5, 6iii), and accurate evaluation of NMJ structure (Figure 1, 2, 3, 4, 5, 6iv; Sleigh *et al*., 2014a).

**Figure 6:**
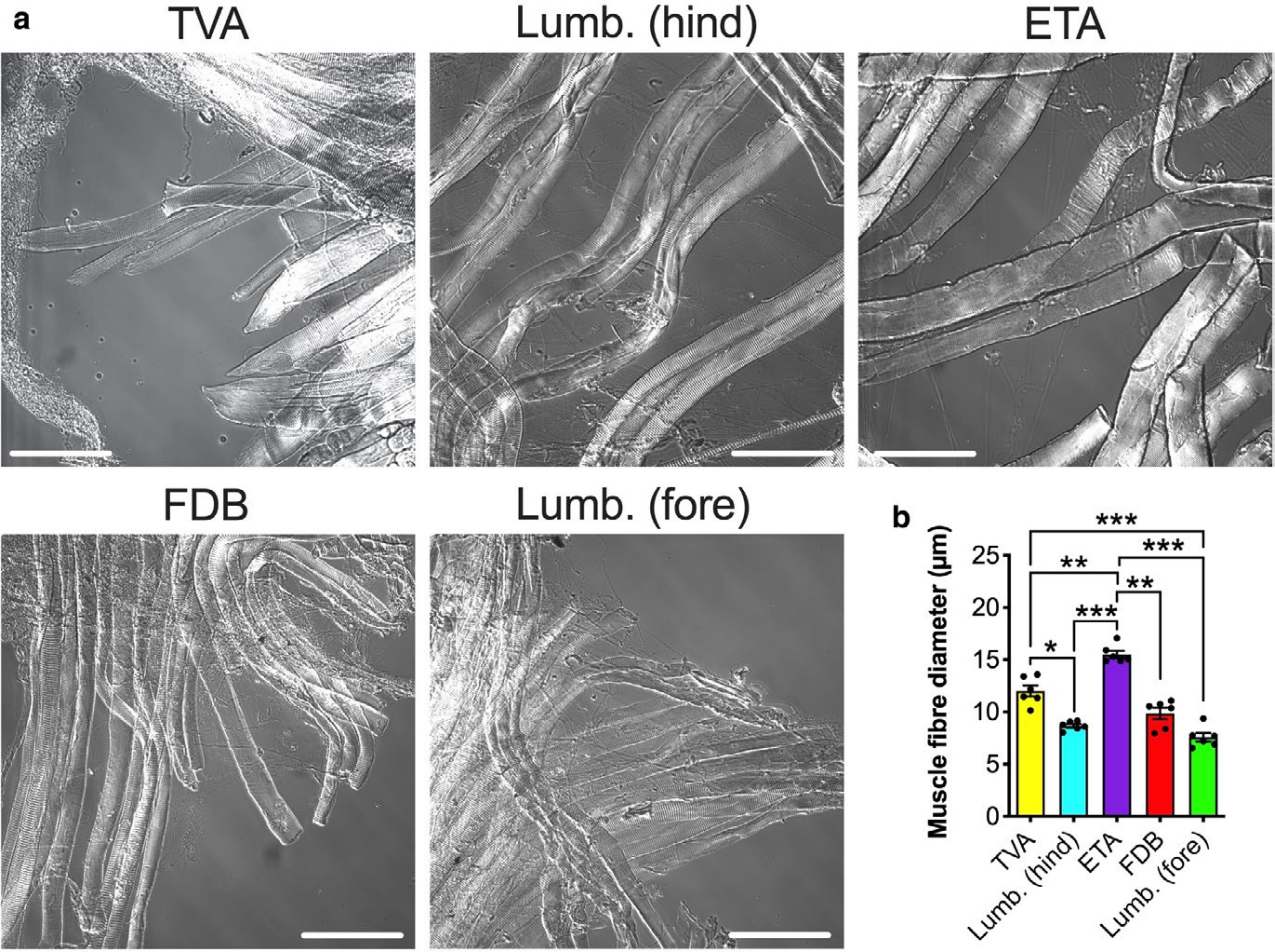
Fibre diameter differs between mature muscles, but does not influence neuromuscular junction (NMJ) morphology. (a) Representative differential interference contrast images of teased muscle fibres from the five wholemount muscles. Scale bars: 100 µm. (b) Fibre diameters are not equal across muscles; however, no significant correlation between fibre diameter and any of the 20 NMJ morphological variables was observed (Table S7). *P* < 0.001 repeated‐measures one‐way anova. ^*^ *P* < 0.05, ^**^ *P* < 0.01, ^***^ *P* < 0.001 Bonferroni's multiple comparisons test. Means ± *SEM* are plotted (*n* = 6), as well as individual data points generated from each mouse (i.e. the mean values from 30 fibres). *Lumb. (fore)*, lumbricals of the forepaw; *Lumb. (hind)*, lumbricals of the hindpaw. See also Figure S3 and Table S7 [Colour figure can be viewed at wileyonlinelibrary.com]

### 3.2 Mature post‐synaptic neuromuscular junction morphology varies more than pre‐synaptic between muscles

Muscles were dissected from wild‐type mice aged P31−32, a timepoint chosen to represent a mature, early adult NMJ. To confirm this, NMJ development was assessed by calculating the percentage of synapses innervated by more than one motor neuron (i.e. poly‐innervation), which is a key feature of immature NMJs (Sanes and Lichtman, 1999). As expected, all muscles had undergone the process of synapse elimination to become mono‐innervated (Table 2), while post‐synapses displayed clear pretzel‐like structures (Figures 1, 2, 3, 4, 5, 6iv and 2a). The foetal to adult AChR subunit switch is also usually complete by this stage (Missias *et al*., 1996). Together, this suggests that analysis at P31−32 allows assessment of relatively well‐developed NMJs across all five muscles.

**Table 2:** P31−32 NMJ morphological variables

Variable	Mean	Maximum	Minimum	Fold difference
Poly‐innervation (%)	0.30	0.50	0.17	2.94
Nerve terminal perimeter (µm)	203.80	270.39	147.61	1.83
Nerve terminal area (µm^2^)	146.62	169.33	120.11	1.41
# Terminal branches	31.32	44.09	19.98	2.21
# Branch points	25.22	28.84	20.80	1.39
Total branch length (µm)	106.28	129.27	82.31	1.57
Average branch length (µm)	4.61	6.03	3.80	1.59
Complexity	4.74	4.97	4.37	1.14
Axon diameter (µm)	1.48	1.64	1.29	1.27
AChR perimeter (µm)	164.56	222.39	118.45	1.88
AChR area (µm^2^)	198.64	254.43	150.07	1.70
Endplate diameter (µm)	27.14	35.28	22.92	1.54
Endplate perimeter (µm)	74.04	92.07	62.22	1.48
Endplate area (µm^2^)	296.98	407.30	214.33	1.90
# AChR clusters	3.06	4.25	1.98	2.15
AChR cluster area (µm^2^)	101.52	114.73	83.94	1.37
Compactness (%)	68.57	71.02	64.60	1.10
Fragmentation	0.43	0.58	0.31	1.86
Synaptic contact area (µm^2^)	123.34	139.75	102.20	1.37
Overlap (%)	63.36	68.57	56.11	1.22

Mean values for all NMJ variables were calculated for each muscle per animal from 20 NMJs, the means of which (*n* = 6) were then recorded for each muscle. The plotted mean, maximum and minimum values for each NMJ variable were determined from these calculated values. The fold difference was calculated by dividing the maximum value by the minimum. Variables are divided into pre‐synaptic at the top, post‐synaptic in the middle, and combined pre‐ and post‐synaptic at the bottom. See also Figure 2 and Table S2.

AChR, acetylcholine receptor.

NMJ‐morph was then used to assess pre‐synaptic, post‐synaptic and overlapping features of NMJ morphology (Figure 2; Tables 2 and S2). As a relative measure of the range in morphology observed between muscles, the fold difference between the maximum and minimum values for each variable were calculated (Table 2). Assessing inter‐muscle variability in the pre‐synapse, the number of terminal branches showed the greatest fold difference (2.21), while complexity displayed the least (1.14). In the post‐synapse, the number of AChR clusters showed the greatest fold distinction (2.15) and compactness the smallest (1.10). In the two combined pre‐ and post‐synaptic variables of synaptic contact area and overlap, maximum to minimum fold differences of 1.37 and 1.22 were observed, respectively.

As 20 separate repeated‐measures anovas were conducted, the Bonferroni correction was applied to determine that a *P‐*value of less than 0.00256 for each test is required to maintain an *α* of 0.05. With this stringent *P‐*value, no significant differences were observed in pre‐synaptic or combined morphological variables (Table S2). The lack of difference in the overlap between pre‐ and post‐synaptic staining indicates that despite morphological variety between muscles, the level of nerve to muscle interaction remains fairly constant, suggesting that pathological denervation assessed in different muscles can be reliably compared. In contrast, six post‐synaptic variables displayed significant differences between muscles (Figure 2b). The TVA had the largest reported values for most post‐synaptic variables and forepaw lumbricals the smallest. These data indicate that mature NMJs found in a diverse set of wholemount muscles display significant variation in post‐synaptic, but not pre‐synaptic, architecture.

For efficiency, 20 NMJs per muscle per timepoint were analysed rather than the suggested 30 (Jones *et al*., 2016). Nonetheless, graphical evidence is provided that there is little to no distinction in NMJ variables when 20–50 NMJs were observed (Jones *et al*., 2016). Consistent with this, intra‐muscular morphological variation between the six mice dissected here was low. This is also pertinent to the point that several paw muscles were combined in the presented analyses, because subtle distinctions in morphologies have been reported between the first and fourth lumbricals of the hindpaw and the second and fourth lumbricals of the forepaws (Jones *et al*., 2016). Muscles were combined because comparative denervation analyses have already been performed in this manner in mouse models of neuromuscular disease (Sleigh *et al*., 2014b; 2020).

### 3.3 Intra‐muscular variation in synaptic architecture is greater at immature synapses

To determine whether developing NMJs display similar distinctions in NMJ structure, morphological analysis was also performed at P7. Poly‐innervation counts confirmed that the process of synapse elimination was not yet complete in any of the five muscles (Table 3), indicating that the P7 timepoint allows analysis of immature neuromuscular contacts. It has been suggested that synapse elimination generally occurs along a proximal‐to‐distal gradient, with NMJs further from the thorax taking longer to undergo this important developmental process (Bixby and van Essen, 1979). However, the TVA, which is the most proximal muscle assessed here, showed the highest percentage of poly‐innervated NMJs (~36%). Moreover, the three paw muscles showed very similar degrees of poly‐innervation (18%–19%) despite the FDB and hindpaw lumbricals being more distal than forepaw lumbricals. These data indicate that at P7 there is no body‐wide location effect on synapse elimination. Nonetheless, regional distinctions in poly‐innervation have been reported (Bixby and van Essen, 1979), so perhaps additional factors, such as muscle fibre type (Lee, 2019), contribute to the observed results (see below).

**Table 3:** P7 NMJ morphological variables

Variable	Mean	Maximum	Minimum	Fold difference
Poly‐innervation (%)	19.90	35.50	8.83	4.02
Nerve terminal perimeter (µm)	96.92	142.96	52.91	2.70
Nerve terminal area (µm^2^)	35.39	52.27	17.63	2.96
# Terminal branches	51.05	78.25	28.57	2.74
# Branch points	35.38	58.31	17.68	3.30
Total branch length (µm)	55.66	85.26	28.80	2.96
Average branch length (µm)	1.31	1.55	1.15	1.35
Complexity	4.68	5.42	3.96	1.37
Axon diameter (µm)	0.84	0.91	0.76	1.20
AChR perimeter (µm)	116.44	142.25	81.63	1.74
AChR area (µm^2^)	66.62	100.06	31.66	3.16
Endplate diameter (µm)	15.48	21.63	12.33	1.75
Endplate perimeter (µm)	42.31	54.55	33.06	1.65
Endplate area (µm^2^)	85.59	121.07	46.33	2.61
# AChR clusters	3.92	4.56	3.02	1.51
AChR cluster area (µm^2^)	25.38	38.70	15.10	2.56
Compactness (%)	76.22	82.94	68.72	1.21
Fragmentation	0.54	0.60	0.47	1.28
Synaptic contact area (µm^2^)	27.77	42.53	11.88	3.58
Overlap (%)	42.11	46.45	37.31	1.24

Mean values for all NMJ variables were calculated for each muscle per animal from 20 NMJs, the means of which (*n* = 6) were then recorded for each muscle. The plotted mean, maximum and minimum values for each NMJ variable were determined from these calculated values. The fold difference was calculated by dividing the maximum value by the minimum. Variables are divided into pre‐synaptic at the top, post‐synaptic in the middle, and combined pre‐ and post‐synaptic at the bottom. See also Figure 3 and Table S3.

AChR, acetylcholine receptor.

The NMJ‐morph software was used to assess pre‐synaptic, post‐synaptic and overlapping morphologies at the developing NMJ (Figure 3; Tables 3 and S3). Unlike at mature synapses, all but two variables, average branch length and axon diameter, showed significant distinctions between muscles at P7 (Figure 3b; Table S3). Similar to several pre‐synaptic features at P31−32, most motor terminal variables for the TVA and ETA were larger than FDB and hindpaw lumbricals, with the forepaw lumbricals showing intermediate values. The number of branch points displayed the greatest fold difference between maximum and minimum values (3.30), while axon diameter displayed the least (1.20; Table 3).

The developing post‐synapse showed a similar number of significant differences between muscles as the mature NMJ, with all variables showing distinctions, except for AChR perimeter, the number of AChR clusters and fragmentation (Figure 3c). Similar to pre‐synaptic morphology, the TVA and ETA generally had the largest post‐synaptic structures, followed by the forepaw lumbricals, then the FDB and hindpaw lumbricals. AChR area showed the greatest fold difference between muscles (3.16), while compactness showed the smallest (1.21; Table 3).

Finally, consistent with the myriad of pre‐ and post‐synaptic distinctions between muscles, the synaptic contact area showed significant variation (Figure 3d), and a fold difference between maximum and minimum values of 3.58 (Table 3). Repeating the finding at P31−32, the percentage overlap between pre‐ and post‐synapse did not differ (1.24‐fold difference), once again confirming the possibility of cross‐muscle denervation analysis.

Together, these analyses of the developing NMJ indicate that there is more variation between muscles at P7 than at the mature adult synapse; the post‐synapse displays a comparably large amount of inter‐muscle variation at both timepoints, while the pre‐synapse shows greater heterogeneity between muscles during immaturity.

### 3.4 Neuromuscular junction endplate area is a powerful discriminator of developmental stage and muscle type

To better understand which morphological variables changed most throughout NMJ development, a PCA was performed. PCA is an exploratory data analysis tool widely used for dimensionality reduction because of its ability to condense large datasets in an interpretable way. The technique functions by collapsing the existing variables into new principal components (PCs) that are created through linear combinations of the original dataset. During the creation of PCs, the variance between components is maximised so that each new component is as different from each other as possible, and thus generates additional information about the original dataset.

Neuromuscular junctions from both P7 and P31−32 were plotted along the two most important PCs (Figure 4). PC1 on the *x*‐axis accounts for 90.5% of the variation in the dataset and clearly separated the two timepoints, with some muscles within timepoints also diverging (e.g. P7 FDB and TVA). PC2 on the *y*‐axis explains only 4.9% of the variation within the dataset, but roughly separated NMJs across different muscles, with little to no separation of timepoints. Distribution of muscles across PC1 and PC2 indicates that at P31−32, the forepaw lumbricals are most different from the TVA, while FDB and hindpaw lumbricals are most similar (Figure 4). This is corroborated by clustering analysis (Figure S1a) and pairwise significance testing between all muscles for all variables (Table S2). At P7, the FDB and hindpaw lumbricals are again most similar, while the TVA and ETA are most dissimilar to the FDB and hindpaw lumbricals (Figure 4). This is again supported by clustering analysis (Figure S1b) and pairwise significance testing (Table S3). Generally speaking, NMJs at maturity were more variable than developing NMJs (greater spread of datapoints), especially along PC2, with the TVA showing the greatest irregularity at P31−32 and the ETA at P7. These patterns were corroborated by the clustering analyses (Figure S1a,b). This variability may be due to the constituent fibres of the imaged muscles; for instance, the TVA, which has the greatest variability at maturity, has the highest percentage of slow twitch fibres (~32%); thus, if a fibre/NMJ is selected at random, it has more chance of being slow‐twitch than in the other muscles. This pattern is not observed at P7, but muscle fibre types are not yet fully determined at this age in mice (Schiaffino and Reggiani, 2011). That being said, the 100% fast twitch forepaw lumbrical muscle shows similar variability at P31−32, if not more, to the remaining three muscles. The variability may thus instead be linked to additional properties of the muscle, for example, differences in fibre or overall muscle sizes.

To find which features contributed most to PC1 and PC2, a loading plot analysis was performed (Figure S1c), which shows how strongly each morphological variable drives each PC. Endplate area added most to PC1, with nerve terminal area, AChR perimeter and AChR cluster area also contributing. The endplate area strongly reflects the overall size of the NMJ because it includes the area contained within the perimeter of α‐BTX staining. Analysis of NMJs in nine different muscles from wild‐type CD1 mice aged 6 weeks corroborates this finding that NMJ size drives the majority of variation between different muscles (Jones *et al*., 2016). The number of terminal branches and AChR perimeter contributed the most to PC2, with much smaller influences from poly‐innervation and compactness.

Finally, an Eigencor plot was created, which depicts Pearson product moment correlation coefficients (*r*) between PCs and the four metadata variables of percentage fast twitch muscle fibres, animal weight, sex and age (Figure S1d). PC1 correlated significantly with age (*P* < 0.001, *r* = 0.87), which is unsurprising as this PC clearly separated developing and mature NMJs, and weight (*P* < 0.001, *r* = −0.84), which is obviously impacted by age (Table S1). Nevertheless, samples within timepoints did not appear to cluster by weight (Figure S1a,b), indicating that small differences in body weight at a particular timepoint do not greatly impact NMJ morphology. Age (*r* = 0.42) and weight (*r* = −0.41) also correlated with PC2 (*P* < 0.05), although less so. However, muscle fibre type correlated most significantly with PC2 (*P* < 0.001, *r* = −0.60), which indicates that fibre type percentage is possibly driving distinctions in NMJ morphology between the assessed muscles. No correlations were observed between sex and PCs, suggesting that up to the age of P31−32, there are no major differences between NMJs of male and female mice, which was corroborated by the clustering analyses (Figure S1a,b). Nonetheless, as the two sexes diverge in muscle mass and strength later in life, this may not continue to be observed.

### 3.5 Morphological change over neuromuscular junction development differs between muscles

As there is more structural variety between muscles at developing NMJs than mature synapses, the rate of maturation across the muscles is likely to differ, such that differences at P7 become smaller by P31−32. Supporting this idea is the observation that synapse elimination does not occur to the same extent by P7 in all five muscles. To assess morphological development and growth at the NMJ, the percentage change over time was calculated for each muscle, and two‐way anovas performed on all 20 NMJ morphological properties, with age and muscle being the two statistical test variables (Table S4). All morphological variables except for complexity showed a significant difference between ages. There is also a significant difference between muscles for most variables, except for average branch length, axon diameter, AChR cluster area, compactness and overlap. Similarly, individual muscles show differences in most variables between timepoints, with at least one muscle displaying a difference in every variable (Table S4). Additionally, several different pre‐ and post‐synaptic variables show a significant interaction (Figure 5), indicating that the amount of change occurring at the NMJ during post‐natal development is muscle‐specific, i.e. NMJs across different muscles do not develop and grow at the same rate between P7 and P31−32. Indeed, lumbricals of the hindpaw and FDB showed the greatest average change per morphological variable (260% and 303%, respectively), while the ETA (168%) and TVA (170%) the smallest (percentage values include poly‐innervation; Table S4).

These differences in development/growth rate perhaps reflect the function of each muscle and its relative importance during early post‐natal life. The TVA and ETA are the two muscles with the largest NMJs at both timepoints and that display the least change from P7 to P31−32. These two muscles are therefore perhaps some of the most well developed during the first week of life in mice, at least in terms of the size of pre‐ and post‐synaptic structures (keeping in mind that poly‐innervation is rife in the TVA at P7). The TVA has a role in respiration (Mesquita Montes *et al*., 2016), so has a clear need to be functional from birth. Furthermore, feeding behaviours of pups are likely to be more dependent on postural muscles and those that mediate larger movements of the limbs than perhaps those that facilitate finer movements of the paws. Being able to breathe and feed immediately after birth are clearly two of the most important early mouse behaviours, perhaps accounting for why the TVA and ETA muscles appear most well developed. These results indicate that the rates of NMJ development and growth are driven by their functional importance at birth and during the first weeks of life.

### 3.6 Fast twitch fibres are more likely to be innervated by smaller neuromuscular junctions

A possible cause for the observed inter‐muscular differences in NMJ morphology is the relative abundance of fast and slow twitch fibres. Indeed, NMJ size has been previously reported to be related to muscle fibre type (Prakash *et al*., 1996; Wood and Slater, 1997), and PC2, which separated NMJs across muscles, correlated significantly with the percentage of fast twitch muscle fibres (Figure S1d). Thus, all NMJ variables at P7 and P31−32 were correlated with previously reported (Table 1) fast twitch muscle fibre percentages (Table S5).

First, assessing synapse elimination, no correlations were observed between the percentage of fast twitch fibres and poly‐innervation at both timepoints. This contrasts with findings of Lee (2019), who showed a significant correlation between slow twitch fibres and poly‐innervation across EDL, soleus and sternomastoid muscles of P9 wild‐type mice. This was directly corroborated by observations of individual fibres within the soleus muscle indicating a greater degree of poly‐innervation at NMJs on slow fibres (Lee, 2019). The conflict between the results obtained here and the previous study could be due to several reasons, including the use of different timepoints and muscles. Several additional publications found no link between muscle fibre type and synapse elimination, albeit in different species (mouse vs. rabbit) and using different methods (direct vs. indirect; Bixby and van Essen, 1979; Soha *et al*., 1987; Cramer and van Essen, 1995). Nevertheless, assessing neuronal inputs at the NMJ in combination with immunofluorescent fibre typing is a powerful tool to better understand the importance of fibre type on synapse elimination, and should therefore be used in the future to address this within the five wholemount muscles.

Moving on to the influence of fibre type on additional NMJ variables, no significant correlations were observed between the percentage of fast twitch muscle fibres and any of the pre‐synaptic or overlapping morphologies at either timepoint (Table S5). In contrast, a significant correlation was observed between muscle fibre type and endplate diameter at P31−32 (Figure S2). There was an inverse correlation, indicating that smaller endplate diameters are perhaps linked with fast twitch muscle fibres, although this pattern was not observed at P7. Correlation between fibre type percentages and the rate of NMJ development/growth between P7 and P31−32 in all 20 morphological variables was also assessed, but no significant associations were observed (Table S6). This suggests that fibre type has little to no influence on change in NMJ morphology over the first month of post‐natal development. Instead, perhaps one or a combination of the myriad transmembrane receptors, secreted ligands and other trans‐synaptic proteins found at the NMJ are driving these inter‐muscle distinctions, several of which are known to be involved in the developmental process of synapse elimination (English, 2003; Singhal and Martin, 2011).

Consistent with PC2 of the PCA, muscle fibre type may indeed influence aspects of mature NMJ morphology, with smaller endplate diameters being significantly associated with fast twitch fibres (Figure S2). Although all other parameters at both timepoints did not significantly correlate with fibre type, many showed the same general trend as endplate diameters, i.e. larger features of NMJ morphology tended to be observed in muscles with lower percentages of fast twitch fibres, and this was particularly true for the post‐synaptic architecture. There are studies in individual muscles that both support (McArdle and Sansone, 1977; Wood and Slater, 1997) and contrast (Prakash *et al*., 1996) with this finding. Nevertheless, the fibre type percentages used here were drawn from several different published works, which is likely to impact consistency and accuracy across all five muscles. Furthermore, the percentage of fast twitch fibres reported for the TVA was taken from rat, because mouse data were unavailable. Not all muscles show similar slow and fast twitch fibre type percentages between these two rodents, for example, the plantaris is 0% slow twitch in mouse and ~8% in rat, with rat muscles reported to show a relatively ‘slower’ phenotype than mouse muscles (Bloemberg and Quadrilatero, 2012). The 68% fast twitch fibres in the TVA may thus be an underestimate for mice. Nevertheless, several muscles between the two species are comparable, for example, tibialis anterior, EDL and masseter (Schiaffino, 1974; Gorza, 1990; Bloemberg and Quadrilatero, 2012). Furthermore, given that the TVA is at one end of the fibre type spectrum of the muscles analysed in this study, the impact on correlations of the mouse TVA having a different fast twitch percentage from rat may be less than if the TVA was found within the spectrum. In relation to this and the applicability of the findings of this study for human muscles, fibre type percentages are also only infrequently conserved between mice and humans (Schiaffino and Reggiani, 2011), and there are major distinctions in NMJ morphology (Jones *et al*., 2017).

### 3.7 Muscle fibre size does not dictate mature neuromuscular junction morphology

Neuromuscular junction size has been reported to correlate with muscle fibre size in several different species (Harris, 1954; Nyström, 1968; Kuno *et al*., 1971; Balice‐Gordon and Lichtman, 1990; Sieck et al., 2012), which may be contributing to the presented findings. The five wholemount muscles were therefore re‐mounted after teasing so that the influence of muscle fibre diameters on NMJ structure could be assessed (Figure 6a). Only muscles from the P31−32 timepoint were used because the sole morphological correlation with fibre type was identified at that age (Figure S2). Significant differences in fibre diameter between several muscles were observed (Figure 6b), with the ETA having the largest fibres and the forepaw lumbricals the smallest (2.05‐fold difference). When fibre diameters were correlated with the percentage of fast twitch fibres, no significant relationship was found (*P* = 0.483, *r* = −0.419, data not shown). This indicates that fibre type does not impact fibre diameter across the five wholemount muscles that were analysed. This contrasts with the general assumption that fast twitch fibres have larger diameters (Stifani, 2014), and indicates that subsets of muscles may display idiosyncratic fibre features, perhaps linked with overall size and thickness of the muscle, as has been observed previously (Alnaqeeb and Goldspink, 1987).

To determine whether fibre size influences synaptic architecture, fibre diameters were correlated with all NMJ morphological variables at P31−32 (Table S7). No significant associations were observed, suggesting that fibre diameter does not play a major role in determining synaptic structure, with the caveat that individual measured fibres were not the same as those from which NMJs were studied. Similar to the poly‐innervation phenotype, future work is therefore needed to immunohistochemically co‐assess fibre types, their diameters and associated NMJ morphologies within the five muscles to address this more directly. This will be difficult to perform for all fibre subtypes, as many MHC‐specific antibodies work only on non‐fixed muscles (Rossor *et al*., 2020), while fixed samples are needed for SV2/2H3 staining; that being said, co‐staining of fixed muscle with type I and IIa fibre markers has been reported (Sieck et al., 2012; Lee, 2019).

When normalised to muscle fibre size, it has been shown that slow twitch NMJs in rat diaphragm muscle cover a relatively greater area than fast twitch NMJs (Prakash *et al*., 1995b, 1996; Prakash and Sieck, 1998). To determine whether a similar pattern is observed in mice, the ratios of P31−32 endplate diameters and endplate areas to fibre diameters were calculated for each muscle and correlated to fibre type (Figure S3). These two morphological variables were chosen because endplate diameter significantly correlated with fibre type, while endplate area drove most NMJ variation in PC1 and is arguably the best proxy for overall NMJ size. While significant differences were observed between muscles (Figure 3a,c), no correlations between fibre type and relative endplate diameter or relative endplate area were observed (Figure S3b,d). This suggests that the pattern of NMJ size to fibre type ratio observed in rat diaphragm is not replicated in these mouse muscles.

## 4 CONCLUSION

Using an ImageJ‐based workflow, morphometric data on mouse NMJs from five different wholemount muscles were generated at early post‐natal and adult timepoints. Developing NMJs were shown to have much greater inter‐muscle variability in pre‐ and post‐synaptic structures than mature NMJs, which only showed distinctions in their post‐synapse. These differences appear to be independent of muscle fibre diameter, at least at P31−32. Moreover, NMJ growth was significantly different between muscles over this early post‐natal period. It is well known that NMJ size and morphology are predictive of function, and that muscle fibre types differ in their functional requirements of neurotransmission (Kuno *et al*., 1971; Harris and Ribchester, 1979; Waerhaug and Lømo, 1994; Ribchester *et al*., 2004; Jones *et al*., 2016). Hence, NMJ morphologies at both timepoints were correlated with fast twitch muscle fibre percentages. Pre‐synaptic neuronal structures did not associate with fibre type; however, there was a possible link between post‐synaptic endplate diameter and fast twitch fibre percentage. In the future, it will be important to independently verify the fibre type composition of the muscle analysed here and to extend these analyses into additional wholemount and thicker muscles of varying sizes, including smaller predominantly slow twitch muscles and larger mainly fast twitch muscles. This study also provides useful, non‐biased, baseline data on the pre‐ and post‐synaptic morphology of mouse NMJs in five anatomically and functionally diverse muscles at two different timepoints. These data can be used by the scientific community in future cross‐study, comparative work assessing pathological changes occurring at the NMJ in mouse models of neuromuscular disease.

## CONFLICT OF INTERESTS

The authors have no competing interest to declare.

## AUTHOR CONTRIBUTIONS

JNS conceived the experiments; AMM, JNS performed the research; AMM, ALB, JNS analysed the data; GS provided expertise and discussion; AMM, JNS wrote the manuscript with input from all authors. All authors approved submission of this work.

## Supporting information


**Supplementary Material**

## Data Availability

The data that support the findings of this study are available from the corresponding author upon reasonable request.
