# Supplementary material for: Morphological variability is greater at developing than mature mouse neuromuscular junctions
Source: J Anat. 2020 Jun 13;237(4):603–17. doi: 10.1111/joa.13228 (PMC7495279; doi:10.1111/joa.13228)
Supplement: Supplementary file 1 — Supplementary Material [file JOA-237-603-s001.pdf]

Supplementary Figures

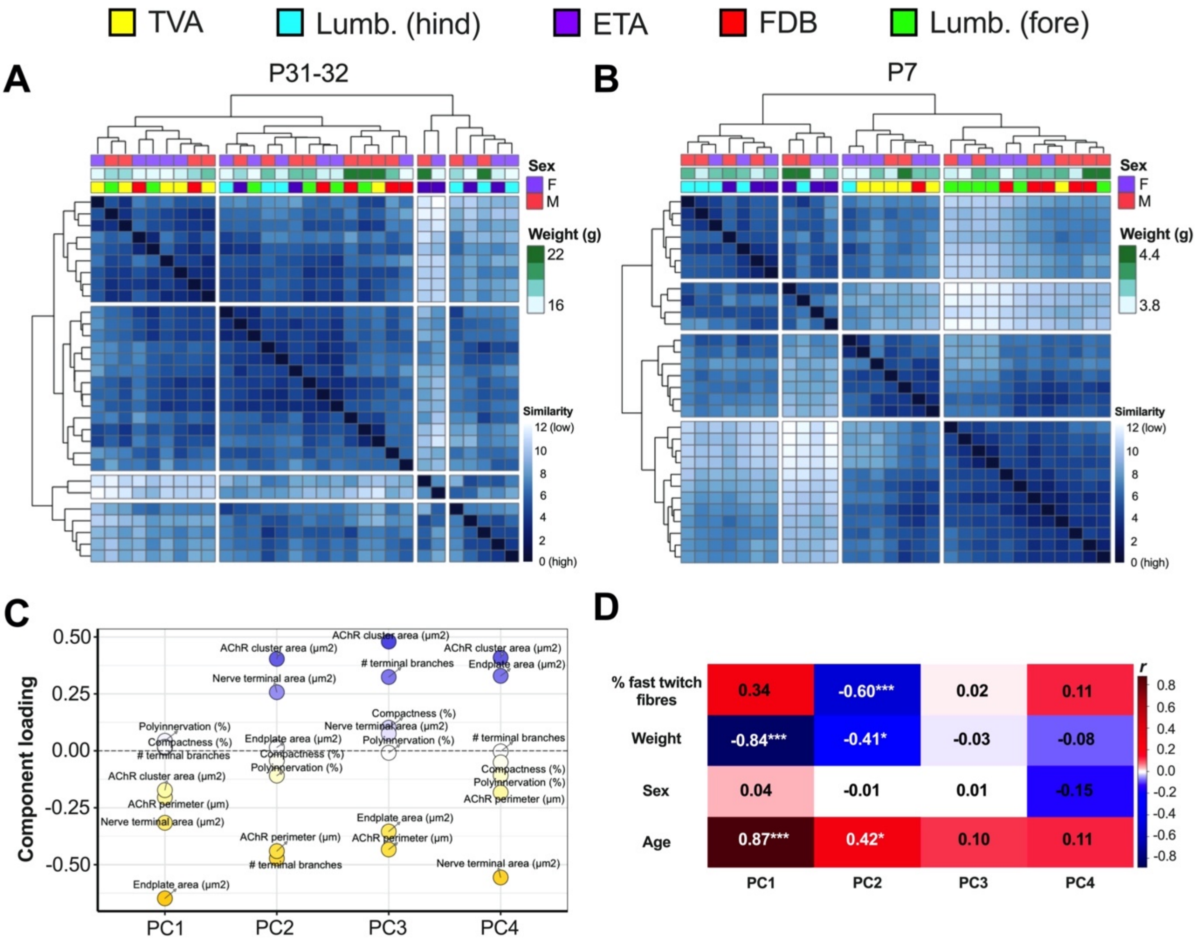

Supplementary Figure 1. Clustering and Principal components analyses of NMJ morphology. (**A**, **B**) Heat maps generated by clustering analyses showing the sample similarly at P31-32 (A) and P7 (B) based on Euclidian distance. The dendrogram and breaks in the heat map represent hierarchal clustering of samples into k = 5 clusters by complete clustering. Each row and column represent a single muscle sample (legend above both panels) with the intensity of blue representing similarity between samples. The vertical branch lengths in the dendrogram give the distance between clusters. At P31-32, the TVA muscle is the most scattered throughout the cluster, with two of the TVA samples even forming a distinct cluster by themselves, suggesting that they are the most variable at this time point (A). At P7, most of the TVA and ETA muscles split off in the first branch in the clustering (B), indicating that these two muscles are most dissimilar from the other three. Note the lack of sample clustering based on sex and body weight at both timepoints. (**C**) PCA loadings plot identifying the morphological features

that contribute most to PC1-PC4. Note that endplate area, nerve terminal area and AChR perimeter drive separation in PC1, whereas the number of terminal branches and AChR perimeter contribute most to PC2. PC3 and PC4 contribute little to the variability between samples (data not shown). (**D**) Eigencor plot identifying significant correlations between PC1-PC4 and metadata variables of '% fast twitch fibres', 'weight', 'sex', and 'age'. Pearson's product moment correlation is reported on the plot, with $r > 0$ shaded red and $r < 0$ shaded blue. $^{*}P < 0.05$, $^{***}P < 0.001$ Bonferroni correction-adjusted P values. See also **Figure 4**.

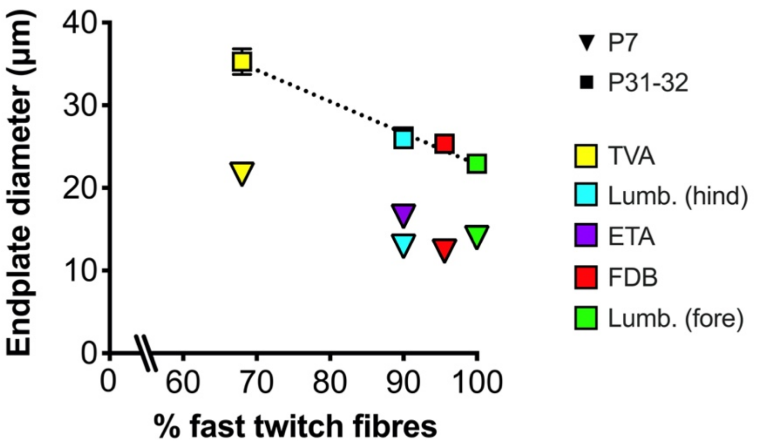

Supplementary Figure 2. Only mature NMJ endplate diameter significantly correlates with muscle fibre type. Correlation was assessed between the percentage of fast twitch muscle fibres of the five dissected muscles (see **Table 1**) and all 20 NMJ morphological variables from both P7 (inverted triangles) and P31-32 (squares) timepoints. Correlation was assessed by calculating Pearson's product moment correlation coefficient (*r*), the results of which are presented in **Supplementary Table 5** along with associated *P* values. Only the post-synaptic variable of endplate diameter significantly correlated with fast twitch fibre percentage ($P < 0.001$), suggesting that smaller NMJs may innervate fast twitch fibres, at least in the five muscles analysed. Means ± standard error of the mean are plotted for the endplate diameters ($n = 6$). *Lumb. (fore)*, lumbricals of the forepaw; *Lumb. (hind)*, lumbricals of the hindpaw. See also **Supplementary Table 5**.

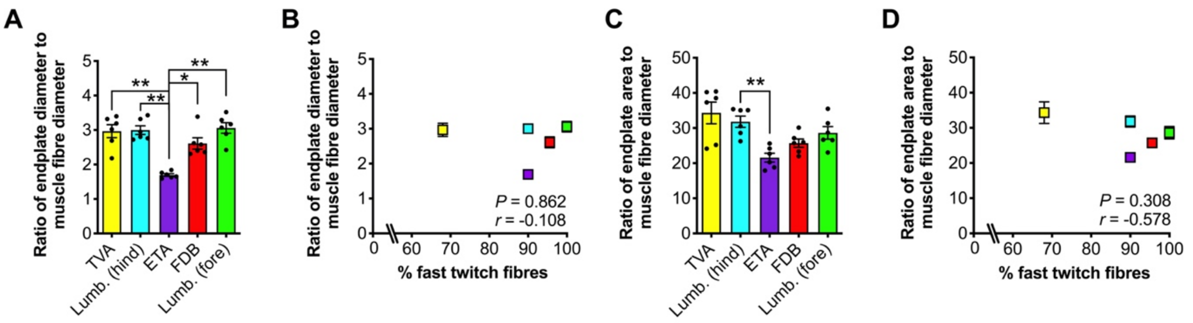

Supplementary Figure 3. The ratios of endplate diameter and endplate area to muscle fibre diameter differ between muscles at P31-32, but do not correlate with fibre type. (**A**, **C**) The five wholemount muscles show different ratios of endplate diameter (A, $P < 0.001$) and endplate area (C, $P = 0.002$) to muscle fibre diameter (repeated-measures one-way ANOVA). $^{*}P < 0.05$, $^{**}P < 0.01$ Bonferroni's multiple comparisons test. Means ± standard error of the mean are plotted ($n = 6$), as well as individual data points generated from each individual mouse. *Lumb. (fore)*, lumbricals of the forepaw; *Lumb. (hind)*, lumbricals of the hindpaw. (**B**, **D**) There is no correlation between the percentage of fast twitch fibres found in each muscle (see **Table 1**) and the ratio of endplate diameter (B, $P = 0.862$, $r = -0.108$) or endplate area (D, $P = 0.308$, $r = -0.578$) to muscle fibre diameter (Pearson's product moment correlation). The colour-coding of muscles is maintained from panels A and C. See also **Figure 6**.

Supplementary Tables

Age	Mouse #	Sex	Weight (g)
P7	1	M	4.5
P7	2	M	4.1
P7	3	M	3.7
P7	4	F	3.6
P7	5	F	4.0
P7	6	F	4.0
P31	1	M	18.3
P32	2	M	22.5
P32	3	M	16.9
P31	4	F	14.5
P31	5	F	14.7
P32	6	F	15.2

Supplementary Table 1. Details of mice used in this study. *M*, male; *F*, female.

Variable	ANOVA *P* value	TVA vs. Lumb. (hind)	TVA vs. ETA	TVA vs. FDB	TVA vs Lumb. (fore)	Lumb. (hind) vs. ETA	Lumb. (hind) vs. FDB	Lumb. (hind) vs Lumb. (fore)	ETA vs. FDB	ETA vs. Lumb. (fore)	FDB vs. Lumb. (fore)
Polyinnervation (%)	0.696	-	-	-	-	-	-	-	-	-	-
Nerve terminal perimeter (μm)	0.003	*ns*	*ns*	*ns*	*ns*	*ns*	*ns*	*	*ns*	*	*ns*
Nerve terminal area (μm^2)	0.079	-	-	-	-	-	-	-	-	-	-
# terminal branches	0.090	-	-	-	-	-	-	-	-	-	-
# branch points	0.387	-	-	-	-	-	-	-	-	-	-
Total branch length (μm)	0.015	*ns*	*ns*	*ns*	*ns*	*ns*	*ns*	*ns*	*ns*	*ns*	*ns*
Average branch length (μm)	0.250	-	-	-	-	-	-	-	-	-	-
Complexity	0.062	-	-	-	-	-	-	-	-	-	-
Axon diameter (μm)	0.120	-	-	-	-	-	-	-	-	-	-
AChR perimeter (μm)	**<0.001$^{\#}$**	*ns*	*ns*	*ns*	*	***	*ns*	*	*ns*	**	*ns*
AChR area (μm^2)	**<0.001$^{\#}$**	*ns*	*ns*	*	**	*ns*	*ns*	*ns*	*ns*	*	*ns*
Endplate diameter (μm)	**<0.001$^{\#}$**	*	*	**	**	*ns*	*ns*	*ns*	*ns*	**	*ns*
Endplate perimeter (μm)	**<0.001$^{\#}$**	*	*ns*	**	**	*ns*	*ns*	*	*ns*	**	*ns*
Endplate area (μm^2)	**<0.001$^{\#}$**	*ns*	*ns*	*	**	*	*ns*	*ns*	*ns*	**	*ns*
# AChR clusters	0.003	*ns*	*ns*	*ns*	*	*ns*	*ns*	*ns*	*ns*	*ns*	*ns*
AChR cluster area (μm^2)	0.046	*ns*	*ns*	*ns*	*ns*	*ns*	*ns*	*ns*	*ns*	*ns*	*ns*
Compactness (%)	0.004	*ns*	*ns*	*ns*	*ns*	**	*ns*	*ns*	*ns*	*ns*	*ns*
Fragmentation	**0.001$^{\#}$**	*ns*	*ns*	*	*	*ns*	*ns*	*ns*	*ns*	*ns*	*ns*
Synaptic contact area (μm^2)	0.117	-	-	-	-	-	-	-	-	-	-
Overlap (%)	0.141	-	-	-	-	-	-	-	-	-	-
	Total sig.	2	1	5	7	3	0	3	0	6	0

Supplementary Table 2. Statistical testing of P31-32 NMJ morphological variables. Pre-synaptic variables are shaded green, post-synaptic variables shaded purple, and combined pre- and post-synaptic variables are unshaded. $^{\#}P < 0.00256$ repeated-measures one-way ANOVA, *i.e.* the Bonferroni correction-adjusted *P* value for an α of 0.05 when performing 20 associated tests. *ns*, not significant; $^{*}P < 0.05$, $^{**}P < 0.01$, $^{***}P < 0.001$ Bonferroni's multiple comparisons test. When the ANOVA $P > 0.05$, Bonferroni's multiple comparisons test was not performed (-). The bottom row shows the total number of morphological variables that were significantly different between each pair of muscles. *Lumb. (fore)*, lumbricals of the forepaw; *Lumb. (hind)*, lumbricals of the hindpaw; *Total sig.*, total significant. See also **Figure 2** and **Table 2**.

Variable	ANOVA *P* value	**TVA vs. Lumb. (hind)**	**TVA vs. ETA**	**TVA vs. FDB**	**TVA vs Lumb. (fore)**	**Lumb. (hind) vs. ETA**	**Lumb. (hind) vs. FDB**	**Lumb. (hind) vs Lumb. (fore)**	**ETA vs. FDB**	**ETA vs. Lumb. (fore)**	**FDB vs. Lumb. (fore)**
Polyinnervation (%)	**<0.001**#	*	**	*	*	*	*ns*	*ns*	*	*ns*	*ns*
Nerve terminal perimeter (µm)	**<0.001**#	**	*ns*	**	*ns*	**	*ns*	*ns*	***	*ns*	*ns*
Nerve terminal area (µm^2)	**<0.001**#	**	*ns*	**	*ns*	***	*ns*	*ns*	***	*	*
# terminal branches	**<0.001**#	*	*ns*	***	*	*	*ns*	*ns*	*	*ns*	*ns*
# branch points	**<0.001**#	*	*ns*	**	*	*	*ns*	*ns*	**	*ns*	*ns*
Total branch length (µm)	**<0.001**#	**	*ns*	**	*	**	*ns*	*ns*	***	*ns*	*ns*
Average branch length (µm)	0.089	-	-	-	-	-	-	-	-	-	-
Complexity	**<0.001**#	**	*ns*	***	**	**	*ns*	*ns*	**	*ns*	*ns*
Axon diameter (µm)	0.300	-	-	-	-	-	-	-	-	-	-
AChR perimeter (µm)	0.007	*	*ns*	**	*ns*	*ns*	*ns*	*ns*	*	*ns*	*ns*
AChR area (µm^2)	**<0.001**#	**	*ns*	***	**	**	*ns*	*ns*	***	**	**
Endplate diameter (µm)	**<0.001**#	**	*ns*	**	**	**	*ns*	*ns*	*	*	*ns*
Endplate perimeter (µm)	**<0.001**#	**	*ns*	**	**	**	*ns*	*ns*	**	*	*
Endplate area (µm^2)	**<0.001**#	**	*ns*	***	*	**	*ns*	*ns*	***	*	*
# AChR clusters	0.057	-	-	-	-	-	-	-	-	-	-
AChR cluster area (µm^2)	**<0.001**#	***	*ns*	***	**	*ns*	*ns*	*ns*	*	*ns*	*ns*
Compactness (%)	**<0.001**#	***	*ns*	**	*ns*	**	*ns*	*ns*	*	*ns*	*ns*
Fragmentation	0.029	*ns*	*ns*	*ns*	*ns*	*ns*	*ns*	*ns*	*ns*	*ns*	*ns*
Synaptic contact area (µm^2)	**<0.001**#	***	*ns*	***	*	***	*ns*	*ns*	***	*	*
Overlap (%)	0.062	-	-	-	-	-	-	-	-	-	-
	Total sig.	15	1	15	11	13	0	0	15	6	5

Supplementary Table 3. Statistical testing of P7 NMJ morphological variables. Pre-synaptic variables are shaded green, post-synaptic variables shaded purple, and combined pre- and post-synaptic variables are unshaded. $^{\#}P < 0.00256$ repeated-measures one-way ANOVA, *i.e.* the Bonferroni correction-adjusted *P* value for an α of 0.05 when performing 20 associated tests. *ns*, not significant; $^{*}P < 0.05$, $^{**}P < 0.01$, $^{***}P < 0.001$ Bonferroni's multiple comparisons test. When the ANOVA $P > 0.05$, Bonferroni's multiple comparisons test was not performed (-). The bottom row shows the total number of morphological variables that were significantly different between each pair of muscles. *Lumb. (fore)*, lumbricals of the forepaw; *Lumb. (hind)*, lumbricals of the hindpaw; *Total sig.*, total significant. See also **Figure 3** and **Table 3**.

	Two-way ANOVA *P* values			TVA		Lumb. (hind)		ETA		FDB		Lumb. (fore)	
**Variable**	**Age**	**Muscle**	**Interaction**	**P31-32 as % of P7**	***P* value**	**P31-32 as % of P7**	***P* value**	**P31-32 as % of P7**	***P* value**	**P31-32 as % of P7**	***P* value**	**P31-32 as % of P7**	***P* value**
Polyinnervation (%)	**<0.001#**	**<0.001#**	**<0.001#**	0	***	3	***	4	***	1	***	2	***
Nerve terminal perimeter (μm)	**<0.001#**	**<0.001#**	0.016	189	***	278	***	180	***	331	***	165	**
Nerve terminal area (μm^2)	**<0.001#**	**<0.001#**	0.198	324	***	619	***	341	***	745	***	340	***
# terminal branches	**<0.001#**	**<0.001#**	0.013	57	**	76	*ns*	50	***	94	*ns*	56	*ns*
# branch points	**<0.001#**	**<0.001#**	**<0.001#**	45	***	115	*ns*	58	**	133	*ns*	73	*ns*
Total branch length (μm)	**<0.001#**	**<0.001#**	0.033	152	***	271	***	157	***	328	***	169	**
Average branch length (μm)	**<0.001#**	0.129	0.401	300	**	383	***	332	**	345	**	389	***
Complexity	0.520	**<0.001#**	**<0.001#**	92	*ns*	112	*ns*	94	*ns*	118	**	97	*ns*
Axon diameter (μm)	**<0.001#**	0.153	0.265	177	***	187	***	199	***	171	***	148	**
AChR perimeter (μm)	**<0.001#**	**<0.001#**	0.008	156	***	153	*	135	*	180	***	97	*ns*
AChR area (μm^2)	**<0.001#**	**<0.001#**	**0.001#**	254	***	430	***	221	***	560	***	259	***
Endplate diameter (μm)	**<0.001#**	**<0.001#**	0.010	163	***	200	***	159	***	206	***	164	***
Endplate perimeter (μm)	**<0.001#**	**<0.001#**	**0.001#**	169	***	196	***	159	***	207	***	157	***
Endplate area (μm^2)	**<0.001#**	**<0.001#**	**<0.001#**	339	***	444	***	275	***	546	***	274	***
# AChR clusters	**<0.001#**	**<0.001#**	0.007	104	*ns*	73	*ns*	94	*ns*	77	*ns*	43	***
AChR cluster area (μm^2)	**<0.001#**	0.527	**<0.001#**	242	***	600	***	250	***	752	***	500	***
Compactness (%)	**<0.001#**	0.015	**<0.001#**	78	***	97	*ns*	81	***	103	*ns*	94	*ns*
Fragmentation	**<0.001#**	**<0.001#**	0.007	106	*ns*	74	*ns*	93	*ns*	71	*ns*	52	***
Synaptic contact area (μm^2)	**<0.001#**	**<0.001#**	0.255	329	***	723	***	336	***	928	***	389	***
Overlap (%)	**<0.001#**	0.099	0.091	126	*	169	***	147	***	168	***	148	***
			**Mean**/Total sig.	**170**	17	**260**	14	**168**	17	**303**	15	**181**	15

Supplementary Table 4. Statistical testing of P7 and P31-32 NMJ morphological variables. Pre-synaptic variables are shaded green, post-synaptic variables shaded purple, and combined pre- and post-synaptic variables are unshaded. On the left, two-way ANOVA *P* values for differences between age, muscle and their interaction are presented. On the right, the mean values of each morphological variable at P31-32 are presented as a percentage of the mean of the same variable at P7 for all five muscles. The *P* values of Sidak's multiple comparisons testing between P7 and P31-32 for each muscle are also presented. $^{\#}P < 0.00256$ two-way ANOVA interaction, *i.e.* the Bonferroni correction-adjusted *P* value for an α of 0.05 when performing 20 associated tests. *ns*, not significant; $^{*}P < 0.05$, $^{**}P < 0.01$, $^{***}P < 0.001$ Sidak's multiple comparisons test. The bottom row shows the mean percentage change for each muscle in bold, and the total number of morphological variables that were significantly different between timepoints for each muscle. *Lumb. (fore)*, lumbricals of the forepaw; *Lumb. (hind)*, lumbricals of the hindpaw; *Total sig.*, total significant. See also **Figure 5**.

	P7		P31-32	
**Variable**	*r*	**Pearson *P* value**	*r*	**Pearson *P* value**
Polyinnervation (%)	-0.771	0.127	-0.409	0.494
Nerve terminal perimeter (µm)	-0.698	0.190	-0.912	0.031
Nerve terminal area (μm^2)	-0.652	0.233	-0.841	0.075
# terminal branches	-0.713	0.176	-0.860	0.062
# branch points	-0.783	0.117	-0.410	0.494
Total branch length (µm)	-0.726	0.165	-0.871	0.055
Average branch length (µm)	0.288	0.639	0.603	0.282
Complexity	-0.696	0.192	-0.762	0.134
Axon diameter (µm)	-0.198	0.750	-0.516	0.373
AChR perimeter (µm)	-0.548	0.339	-0.929	0.022
AChR area (μm^2)	-0.664	0.222	-0.939	0.018
Endplate diameter (µm)	-0.898	0.038	-0.992	**<0.001**$^{\#}$
Endplate perimeter (µm)	-0.796	0.107	-0.979	0.004
Endplate area (μm^2)	-0.632	0.253	-0.942	0.017
# AChR clusters	-0.064	0.918	-0.770	0.128
AChR cluster area (μm^2)	-0.799	0.105	0.338	0.533
Compactness (%)	-0.686	0.202	0.783	0.117
Fragmentation	-0.008	0.990	-0.782	0.118
Synaptic contact area (μm^2)	-0.643	0.242	-0.806	0.100
Overlap (%)	-0.202	0.744	0.884	0.047

Supplementary Table 5. Statistical testing of correlation between the percentage of fast twitch muscle fibres and NMJ morphological variables at P7 and P31-32. Only mature endplate diameter significantly correlated with muscle fibre type. Pre-synaptic variables are shaded green, post-synaptic variables shaded purple, and combined pre- and post-synaptic variables are unshaded. $^{\#}P < 0.00256$ Pearson's product moment correlation, *i.e.* the Bonferroni correction-adjusted *P* value for an α of 0.05 when performing 20 associated tests. See also **Supplementary Figure 2**.

	P7 to P31-32	
**Variable**	***r***	**Pearson *P* value**
Polyinnervation (%)	0.382	0.526
Nerve terminal perimeter (µm)	0.226	0.715
Nerve terminal area (µm^2)	0.362	0.550
# terminal branches	0.301	0.622
# branch points	0.569	0.317
Total branch length (µm)	0.398	0.507
Average branch length (µm)	0.802	0.103
Complexity	0.471	0.424
Axon diameter (µm)	-0.345	0.569
AChR perimeter (µm)	-0.357	0.555
AChR area (µm^2)	0.326	0.593
Endplate diameter (µm)	0.310	0.612
Endplate perimeter (µm)	0.140	0.823
Endplate area (µm^2)	0.130	0.835
# AChR clusters	-0.809	0.097
AChR cluster area (µm^2)	0.629	0.256
Compactness (%)	0.714	0.175
Fragmentation	-0.875	0.052
Synaptic contact area (µm^2)	0.399	0.506
Overlap (%)	0.699	0.189

Supplementary Table 6. Statistical testing of correlation between the percentage of fast twitch muscle fibres and the percentage change in NMJ morphological variables from P7 to P31-32. No correlations were observed. Pre-synaptic variables are shaded green, post-synaptic variables shaded purple, and combined pre- and post-synaptic variables are unshaded.

	P31-32	
**Variable**	***r***	**Pearson *P* value**
Polyinnervation (%)	0.839	0.076
Nerve terminal perimeter (µm)	0.749	0.145
Nerve terminal area (μm^2)	0.722	0.168
# terminal branches	0.823	0.087
# branch points	0.727	0.164
Total branch length (µm)	0.770	0.128
Average branch length (µm)	-0.836	0.078
Complexity	0.822	0.088
Axon diameter (µm)	0.647	0.238
AChR perimeter (µm)	0.721	0.170
AChR area (μm^2)	0.681	0.206
Endplate diameter (µm)	0.396	0.509
Endplate perimeter (µm)	0.592	0.293
Endplate area (μm^2)	0.697	0.191
# AChR clusters	0.889	0.044
AChR cluster area (μm^2)	-0.807	0.099
Compactness (%)	-0.804	0.101
Fragmentation	0.870	0.055
Synaptic contact area (μm^2)	0.712	0.178
Overlap (%)	-0.577	0.309

Supplementary Table 7. Statistical testing of correlation between muscle fibre diameter and NMJ morphological variables at P31-32. No correlations were observed. Pre-synaptic variables are shaded green, post-synaptic variables shaded purple, and combined pre- and post-synaptic variables are unshaded. See also **Figure 6**.
